# Evaluation of the Information Content for Determining the Vascular Tone Type of the Lower Extremities in Varicose Veins: A Case Study

**DOI:** 10.3390/bios13010096

**Published:** 2023-01-06

**Authors:** Ahmad Hammoud, Alexey Tikhomirov, Andrey Briko, Alexander Volkov, Aida Karapetyan, Sergey Shchukin

**Affiliations:** 1 Department of Medical and Technical Information Technology, Bauman Moscow State Technical University, 105005 Moscow, Russia; 2 Scientific and Educational Medical-Technological Center, Bauman Moscow State Technical University, 105005 Moscow, Russia

**Keywords:** bioimpedance, vascular tone, varicose, massage, pulse wave velocity

## Abstract

The incidence of cardiovascular diseases is continuously increasing around the world. Therefore, the study of new methods for diagnosing cardiovascular diseases is very important. Early diagnosis and evaluation of the effectiveness of treatments are among the most important tasks. In this work, we study changes in vascular compliance and vascular tone of the lower extremities in a patient diagnosed with an early stage of varicose veins. The study is based on recording the bioimpedance signals of the lower extremities and their parts using the Rheo-32 multichannel device. Registration in the monitoring system takes place in two stages: the first in a state of relaxation, and the second after applying a local massage on one of the legs for five minutes. The results indicate a change in the type of vascular tone of the lower extremities after the massage, while the type of vascular tone changes and shifts on average towards the normotonic type. The method proposed in this study makes it possible to quantitatively and qualitatively assess changes in the tone of the vessels of the extremities.

## 1. Introduction

Cardiovascular diseases are the leading cause of death worldwide and among the most common diseases in the general population [1]. The need to find diagnostic methods for cardiovascular diseases is constantly increasing, especially for methods that allow us to diagnose early, and impedance plethysmography is one of them [2].

Impedance plethysmography is used to measure the blood volume changes associated with changes in electrical impedance. This technique can be used to measure blood flow in a limb or finger [3].

Electrical impedance plethysmography (electrical IPG) records the impedance of the lower extremity as the blood volume varies. IPG has been proven to be safe, inexpensive, painless, reliable, and effective in monitoring and diagnosing vascular diseases [4,5,6].

Bioimpedance technology has been used in many studies that aimed to monitor changes in blood flow in the lower extremities under unstable conditions, and quantitative results were obtained using this method [7]. The venous volume present in the lower extremities increases when the venous return from the lower extremities is restricted by a cuff. Because of the increase in blood volume, the electrical conductivity in the lower extremities increases because blood is a good conductor of electricity. In order to assess cardiovascular risk and to estimate the presence of hemodynamic and vascular structural changes, it has been confirmed that a pulse waveform analysis based on bioimpedance is a useful tool [8].

One of the very common diseases in adults that affects the quality of life is varicose veins in the lower extremities. Empirically, local massage has been proven to be very effective in treating the symptoms of this disease and relieving pain [9].

In a methodical and structured manner, a specialized therapist performs with the hands a series of manual manipulations during the massage aimed at mobilizing the soft tissues. The activation of the blood circulation resulting from the massage is temporary and has a role in relieving pain or comforting the patient for a short period [10].

Considering that the patient actually feels comfortable after the massage, as has been proven by experimental studies, in this work we ask whether it is possible to measure the effectiveness of this process.

In order to regulate blood flow to living tissues, soft tissues, and organs, peripheral vascular resistance is regulated by vascular tone [11].

For assessment of the type of vascular tone using IPG signals, the ratio of the amplitudes of the diastolic and systolic waves is studied. A small amplitude of the diastolic wave relative to the systolic (or direct) wave corresponds to a hypertonic tone. If the amplitude of the diastolic wave approaches half the amplitude of the direct wave, then the vascular tone can be considered normotonic.

**Figure 1:**
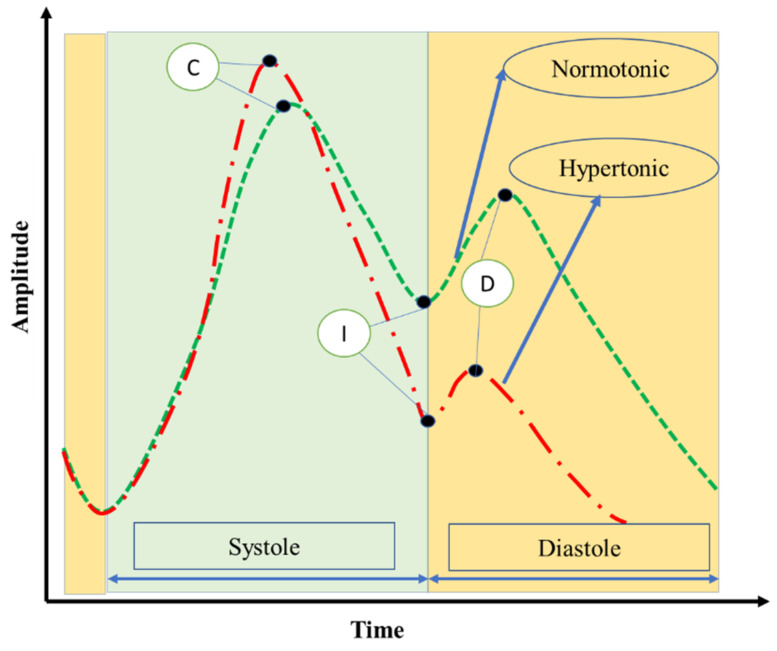
The main forms of IGP signals in the lower extremities. C: peak of the systolic wave, I: incisura, D: peak of the diastolic or reflected wave.

Figure 1 shows the general forms of the IGP signals in the lower extremities, characteristic of signals in healthy subjects, and also highlights the systolic and diastolic phases.

Points C, I, and D express the blood volume change for all vessels for a specific part of the body. This means that changes in the amplitude and time of these points are related to changes in blood volume [12,13]. Therefore, pulsation changes in the bioimpedance signals are mainly due to blood flow changes in the arteries, arterioles, and their branches. In fact, a change in blood flow is related to a change in pressure, which in turn leads to a change in the diameter of blood vessels. Therefore, vascular tone changes can be calculated based on IPG signals.

It has been shown that IPG is sensitive to changes in blood volume in arterial branches up to the sixth branch. In contrast, PWV is primarily used to assess vascular tone in large or major arteries.

Therefore, in our study, we used both IPG and PWV to investigate the evaluation of vascular tone changes in all blood vessels in the studied segment. By combining these two techniques, we were able to assess vascular tone in both small arterial branches and large arteries, providing a more comprehensive understanding of vascular function in the studied extremities [5,7,14,15].

### 1.1. Pulse Wave Velocity

Among markers of arterial disease, arterial stiffness has emerged as an important parameter in estimating cardiovascular risk. Of the various methods used to assess arterial stiffness, the carotid-to-femoral pulse wave velocity (PWV) is relatively easy to determine, has been shown to be reliable, and is the most important. As a matter of fact, it has emerged as the gold standard method as there is a wealth of evidence to support it. It has an association with cardiovascular disease occurring in different populations, and is independent of traditional risk factors [14]. The use of a multichannel bioimpedance system allows us to measure the PWV with some methodological assumptions [15,16]. Using impedance magnitude to estimate pulse transit time (PTT) offers the promise of realizing wearable and cuffless BP devices [17].

### 1.2. Problem Statement

The main objective of this work is to investigate and verify methods for monitoring vascular tone assessment in lower extremities using bioimpedance. In addition, we are interested in monitoring the local femoral pulse wave velocity changes measured through two channels of bioimpedance.

## 2. Materials and Methods

In this study, monitoring was performed using electrical bioimpedance with a multichannel device. This method allows for the monitoring of blood volume changes in the studied section and for several cardiac cycles, in addition to the possibility of obtaining information about the phase of breathing without the use of additional equipment. By using appropriate electrode configurations and recording the signals before and after leg massage, we determined the resulting differences in the signals and analyzed them.

### 2.1. Subject

The subject was a female nonsmoker, 27 years old, BMI = 23.1 (kg/m^2^), with a genetic predisposition in the family to the occurrence of varicose veins. For four years, the subject had annual examinations to detect varicose veins. The measurements were made after confirming the varicose vein in the right leg in the early stages, and the second measurement was made after a five-minute local massage of the right leg.

The medical diagnosis was as follows: “The main trunk of the great saphenous vein is passable, tortuous course and dilatation of the great saphenous vein at the level of the lower leg and thigh: low-phase blood flow valves are insufficient at the level of the lower leg and thigh through the valves of the main trunk of the great saphenous vein. The tributary veins of the large saphenous vein are insolvent, varicose transformed.”

### 2.2. Equipment

The Rheo-32 electrical impedance research system, produced in Moscow, Russia, was utilized in this study. The system boasts several notable characteristics, including:

- 30 precordial electrical impedance channels.
- One transthoracic bioimpedance channel: This channel is specifically designed for measuring bioimpedance through the chest, enabling the assessment of the cardiovascular system and other thoracic tissues.
- One ECG channel.
- A pulse impedance measurement range of −2 to +2 Ohms.
- A channel sampling rate of 500 Hz. This high sampling rate ensures that the measurements are taken quickly and accurately, improving the overall precision of the system.
- The bioimpedance measurement method is tetrapolar.
- The probe current amplitude is 1 mA. A low probe current amplitude minimizes the potential for discomfort and ensures the safety of the measurement process.
- The bandwidth of the bioimpedance channel is 0.01–117 Hz. This wide bandwidth allows for the measurement of a wide range of frequencies, improving the versatility of the system.
- The probing frequency is 100 kHz.

Based on previous and domestic experience in creating multi-channel bioimpedance systems for diagnosing cardiovascular diseases, it is advisable to use probing current frequencies in the range from 50 to 150 kHz. The use of 100 kHz as the probing current frequency in bioimpedance measurements has been found to be both effective and safe, as demonstrated in several studies [18,19].

In addition, this frequency has been shown to provide a signal that is primarily based on changes in resistance (the real component of bioimpedance), with a minimal contribution from reactance (the imaginary component). This is important when studying changes in blood volume using bioimpedance, as the value of bioimpedance should mainly reflect changes in resistance. Using a frequency of 100 kHz has been shown to allow for the determination of up to 90% of the resistance changes associated with a change in blood volume, while minimizing the influence of reactance on the measurement [20,21]. As a result, 100 kHz is a suitable choice for monitoring vascular tone using bioimpedance.

### 2.3. Electrode Configurations

Each electrode was connected to the contact point of the limb symmetrically from two sides. Thus, eight electrodes per channel were required. CERACARTA Italy, Top Trace ECG Electrodes (50 mm) with Ag/AgCl sensors were used for the experiments. In addition to the ECG signal, the signals were recorded from three major segments for each leg; the following channels were for the right leg:

1. Ch1—transthoracic channel;
2. Ch2—right thigh (RLU);
3. Ch3—right leg (RL);
4. Ch4—lower part of the right leg (RLD).

The same was true for the left leg.

**Figure 2:**
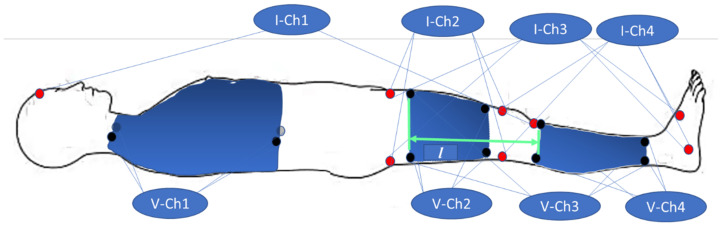
Electrode placement for transthoracic (Ch1), Thigh (Ch2), Whole leg (Ch3), and Lower leg (Ch4).

Measurement points for these signals are shown in Figure 2.

### 2.4. Experimental Procedure

**Figure 3:**
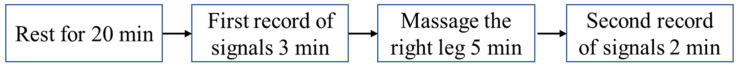
The experimental stages.

For the first step, the subject was lying down and fully relaxed. Recording of the bioimpedance signals began after 20 min of complete relaxation. The signals were recorded for 3 min with free breathing without interruptions. After that, a local massage was performed on the right leg by pressing in an annular manner from the bottom of the foot to the head of the thigh, massaging the muscle areas for a period of 5 min, in order to push the blood stuck in the veins upwards and stimulate blood circulation. Then signals were recorded for 2 min. The experimental stages are presented in Figure 3.

### 2.5. Signal Processing and Breathing Phase Detection

First of all, the signals had to be smoothed. Many methods can be used for smoothing the bioimpedance signal [22]; in this work, we used the Savitsky–Golay method with window width (37–57) and a polynomial order equal to 1.

ECG processing and R-peak detection were performed using the Heartpy library based on [23].

**Figure 4:**
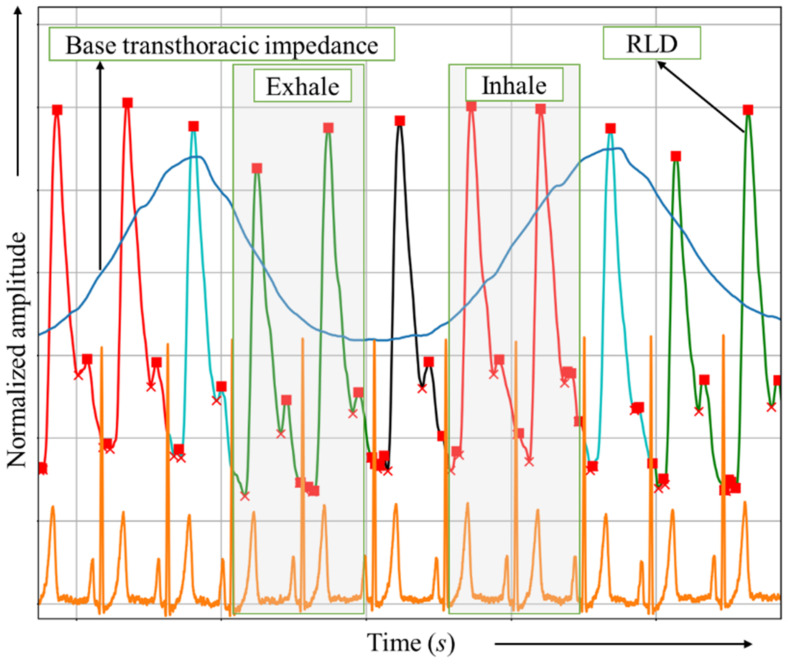
Division of cycles according to the phase of respiration (green—exhale, red—inhale, cyan—end of inhale, black—end of exhale); blue line is the base transthoracic impedance signal, and orange is the ECG signal.

In order to determine the cycles that follow inhalation and exhalation, the transthoracic bioimpedance signal was used [24,25]. At low frequencies of about 10 Hz, the changes in bioimpedance measured in the transthoracic region are mainly related to breathing, as the resistance measured at low frequencies increases steadily with inhalation due to air entering the lungs and decreases in turn when exhaling [25]; this is what can be called base transthoracic bioimpedance, as shown in Figure 4.

**Figure 5:**
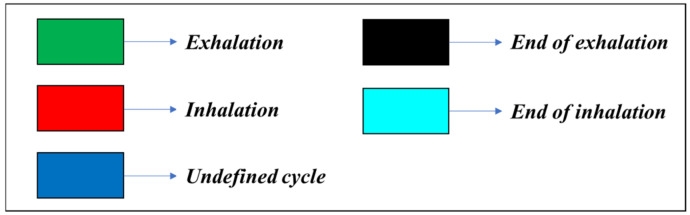
The legend of signal colors.

The legend of the signal colors is presented in Figure 5.

### 2.6. Vascular Tone and Hemodynamic Evaluation

When a pressure wave passes through a blood vessel, it expands, and this expansion is expressed by vascular tone [26]. To identify the type of vascular tone using bioimpedance signals, we utilized two parameters based on the characteristics of the systolic and diastolic or dicrotic waves (as depicted in Figure 1). The parameters we considered were the amplitudes of the systolic wave, diastolic wave, and incisura. The systolic wave originates from the interaction between the volume of blood pumped into the arterial bed and the resistance of this vascular system. The diastolic wave is generated from reflection from the peripheral part of the arterial tree, including the smallest arteries and arterioles. An incisura, resulting from the combination of the systolic and diastolic waves, is formed with a depth dependent on the formation and interaction of these waves. To determine the type of vascular tone, we calculated the diastolic index (DCI), which reflects venous outflow, and the dicrotic index (DKI), which reflects the tone of small vessels, using the following equations:

DKI = AI/AC (1)

DCI = AD/AC. (2)

AC, AI, and AD are the amplitudes of the systolic wave peak, incisura, and diastolic wave peak, respectively. The vascular tone type can be determined based on the DKI–DCI phase plane. This tone has three main types: normotonic, hypotonic, and hypertonic [27]. Detailed information for determining the type of vascular tone using bioimpedance signals can be found in our previous work [22,25,28].

### 2.7. Local Pulse Wave Velocity Measurement

Pulse wave velocity (PWV) is an important parameter in the analysis of arterial tree behavior and a significant marker of cardiovascular risk. It is considered the gold standard for assessment of arterial elastance and is directly related to arterial stiffness (as described in Equation (3)). Measuring PWV is relatively simple, but results can vary depending on the method used. While the desired value can be obtained from either the transit time between two points of the flow or diameter wave (or an artificial perturbation) or the pressure wave over a given distance, it is technically easier to measure pressure than flow or diameter. Most previous studies have only considered the propagation velocity of the invasively measured pressure wave [29,30].

(3) $$PWV=\sqrt{Eh/2R\rho },$$

where *E* is Young’s modulus in the circumferential direction, ρ is the density, and *h* is the wall thickness.

Kusche et al. [15] used a multichannel bioimpedance device to measure the *PWV*, and found an average speed for 20 s of about 7.7 m/s. The measurement was made using traditional methods, where the velocity of the pulse transmission between two points is calculated with a known distance between them.

**Figure 6:**
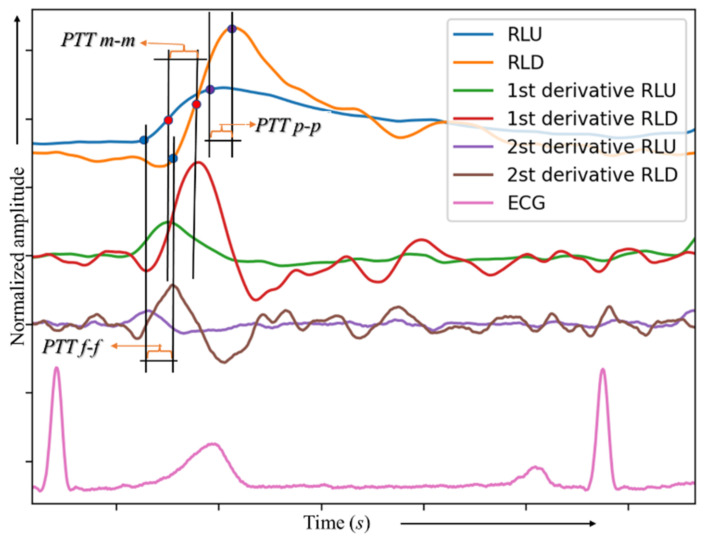
Pulse transit time PTT: peak-to-peak PTT (PTTp-p), middle-to-middle PTT (PTTm-m), and foot-to-foot PTT (PTTf-f).

In Kusche’s study, the time between the two peaks of the diastolic wave was used (PTTp-p; see Figure 6). In this work we used the time between the two inflection points (PTTm-m), as it gives more reliable results than the diastolic wave peak; the time between them, which is known as the filling time, is affected by many factors that are not completely related to the speed of pulse transmission. There is another study underway to monitor changes in blood pressure based on changes in the speed of the pulse wave using an average rate for all measurements (PTTp-p, PTTm-m, and PTTf-f). Figure 6 presents the main measurement methods of PTT.

Considering that the bioimpedance signals express changes in the volume of blood, and in the context of studying the PWV, time (PTTf-f) is the closest to the theory, considering that it expresses the beginning of the change in the volume of blood and thus the beginning of the change in pressure, while the rest of the points are affected by pressure and other factors. For example, we studied the PWV in the arteries, while the bioimpedance signal expresses changes in blood volume not only in the arteries, but in the studied section as a whole, including veins and capillaries as well. Therefore, even horizontal and small electrode combinations will not lead to an accurate calculation of the velocity because, in any case, this impedance will pass through the capillaries and be affected by them. However, with regard to the use of time (PTTf-f), it was not taken into account in this study because in real signals, this point is unstable and the error rate will be very high.

The dependency of *PTT* on *PWV* is given by Equation (4) [31]:

(4) PWV=l/PTT ,

where *l* is the distance between two measurement points (see Figure 1).

## 3. Results

Table 1 presents information about signals recorded before and after the massage. It details the duration of the recording, the number of recorded cycles, the cycles of inhalation and exhalation, the average heart rate, and the respiratory rate.

The recording information shows that the subject was in a state of complete rest during the first recording before the massage and the second after the massage, as the heart rate and breathing rate did not change significantly during the experiment and were in the normal range.

To monitor vascular tone changes, we used two methods:

### 3.1. Vascular Tone for Multi Cycles

First, all the recording cycles are shown where they are located within (DKI, DCI) points, and we could determine the type of vascular tone during the entire recording. Showing points for all cardiac cycles can confuse the display of information due to the large number of cycles, so we show only the points that represent the center of gravity of the points (DKI, DCI) for each phase of respiration.

**Figure 7:**
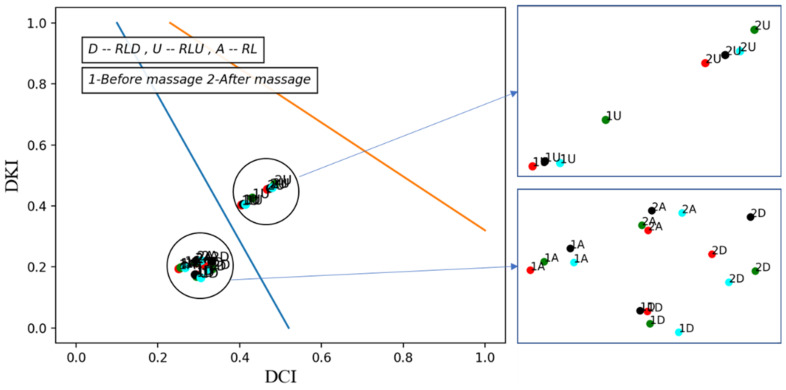
Type of vascular tone of the left leg (massaged) before and after the massage.

**Figure 8:**
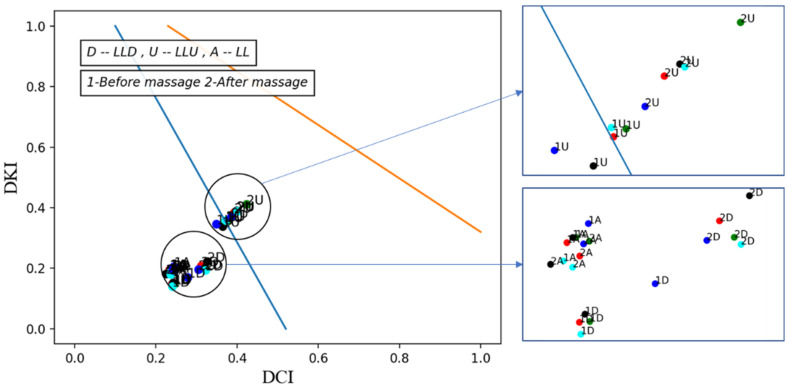
Type of vascular tone of the right leg (non-massaged) before and after the massage.

Variations in vascular tone for each segment are shown before massage (1) and after massage (2), for the right leg (massaged) in Figure 7 and the left leg (non-massaged) in Figure 8.

We notice from Figure 7 and Figure 8 that the type of vascular tone in both legs in the entire leg channel and the lower leg is located in the hypertonic area, but in the recordings after the massage, the points approaching the normatonic were more noticeably in the right leg (massaged).

As for the thigh, the type of vascular tone in general is located in the normotonic area, but on both limbs it is very close in the first recording (before the massage) of the hypertonic area. This indicates the presence of several heart cycles where the vascular tone is within the hypertonic area and, therefore, we used the second method to display changes in vascular tone in the thigh area over time (or successive cardiac cycles).

### 3.2. Local-Time Vascular Tone Changes

**Figure 9:**
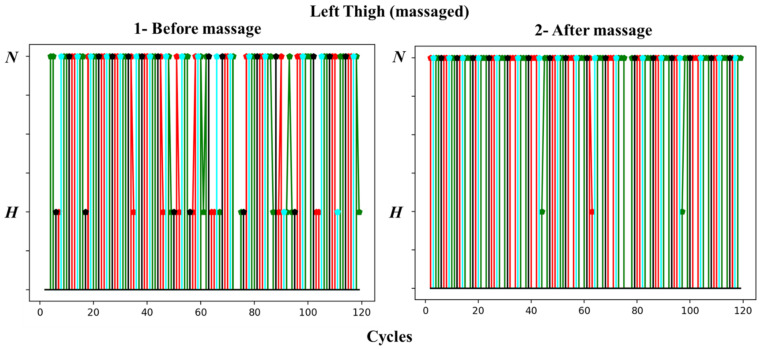
Changes in vascular tone for 120 cardiac cycles in the left thigh (massaged) before and after massage.

**Figure 10:**
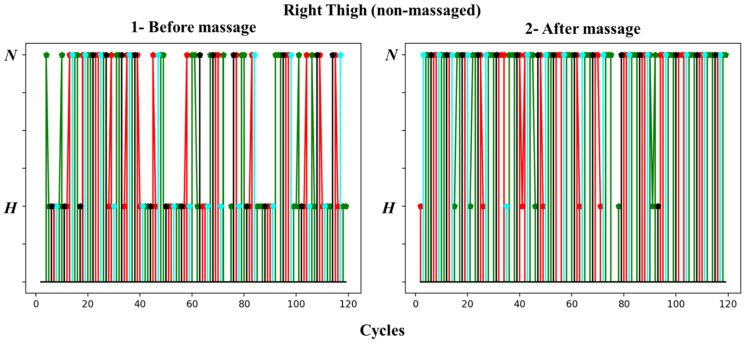
Changes in vascular tone for 120 cardiac cycles in the right thigh (non-massaged) before and after massage.

In Figure 9 and Figure 10, we present the changes in vascular tone for 120 cardiac cycles in the thigh area before and after massage for the left leg and right leg, respectively. The colors of the points are related to the phase of respiration, as shown in Figure 5.

The figures show no pattern of vascular tone changes in either thigh prior to massage. The tone moves from hypertonic to normotonic and then back without a specific rhythm. However, it can be clearly observed that the number of cycles in which the vascular tone is hypertonic in the pre-massage stage is much smaller than after the massage, but in a varying proportion, as the transition to normotonic appears in the massaged thigh.

Many of the cycles following the hypertonic before the massage occurred during the end of the exhalation (black color); we noticed the absence of cycles that followed the end of the exhalation in the recording after the massage.

### 3.3. Pulse Wave Velocity

**Figure 11:**
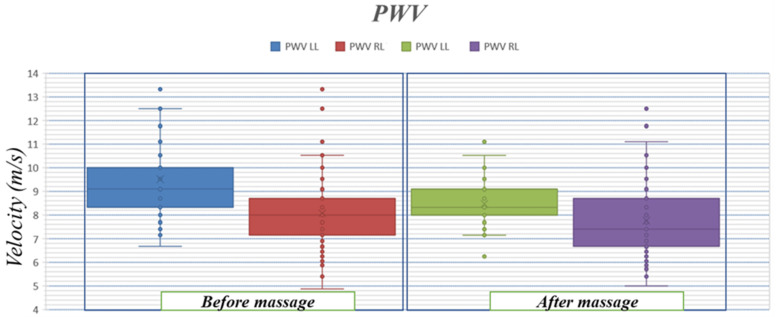
Pulse wave velocity results in the right and left thigh before and after massage.

The velocity of the pulse wave measured in this study is the velocity of the wave in the thigh region. The distance between the first measuring electrode in the thigh and the first measuring electrode in the lower leg is the dimension used to calculate the *PWV* and for the studied subject it is equal to l=40 cm (see Figure 1). The velocity measurement results for the entire recording duration are in Figure 11.

Before the massage, the average speed was 9.2 m/s in the left leg and 8 m/s in the right leg. The measured velocity decreased slightly in both legs after the massage, to 8.4 m/s in the left leg and 7.4 m/s in the right leg. The results show a decrease in the range of the left leg (massaged) after the massage: before the massage it had the ratio [±0.8] and after the massage [±0.6]; for the right leg (non-massaged), the range increased from [±0.8] before the massage to [±1] after the massage, but did not exceed the limits in the stage before the massage. For the massaged leg, the maximum after the massage did not exceed the average value before the massage.

## 4. Discussions

### 4.1. Vascular Tone Changes

The results of changes in the type of vascular tone before and after the massage show the approach of the type of vascular tone after massage to the normotonic area in all studied segments, even if the points were completely located in the hypertonic region (whole leg and lower leg), which means an increase in the wave amplitude in the diastolic stage to the amplitude of the systolic wave.

Studying the temporal changes in the type of vascular tone not only gives information about the general changes in the type of vascular tone, but also allows us to know the extent to which the type of vascular tone has improved by studying the number of cycles in which the vascular tone was hypertonic before and after the massage. As shown in Table 2, the percentage of cycles that were hypertonic decreased in relation to the total number of cycles for the left leg (massaged), from 22.8% before the massage to 2.4% after. The same applies to the right leg, where the percentage decreased from 48.4% before the massage to 11% after.

The results of this study are consistent with [32], in which it was observed that massaging one limb leads to an increase in perfusion in the contralateral limb, but at a lower rate.

The transmission of the change in the type of vascular tone to the opposite end gives an indication that this transmission is due to the neural regulation of the vascular tone [33], but the experimental procedure that we used did not allow us to accurately determine the way and time of transmission, as the recordings were made after the five-minute massage.

### 4.2. Pulse Wave Velocity

The results of *PWV* in this study are close to the values of *PWV* from previous studies [34,35]. The decrease in *PWV* between the recordings before and after the massage indicates a decrease in the arterial tension in the studied area. These results are consistent with the nature of the experiment, as massage leads to an increase in the effectiveness of blood circulation and temporarily relieves vascular tension. Therefore, the use of this technique enables us to evaluate the nature of the procedures used in the temporary or permanent treatment of vascular diseases associated with pulse wave velocity.

### 4.3. Limitations

The methods used in this study depend on the amplitude of the recorded signals for each cardiac cycle, but do not study changes in the overall signal shape. That is, the crest time or width of the systole or diastole wave is not taken into account, and monitoring these indicators requires independent studies.

The capabilities of the device used, especially for the sampling time with 500 Hz, do not allow us to obtain very accurate results regarding the pulse wave velocity. If a similar device is used with a sampling time equal to 1 kHz or more, the results will be more accurate and we can calculate the *PWV* using PTTf-f, but it remains to achieve this frequency for 32 combined channels as the device is designed to be technically difficult.

This study relates to a special case only and its results cannot be generalized in terms of diagnosis. Rather, it can be considered confirmation of the possibility of using the bioimpedance monitoring technique to study local changes in circulatory activity and hemodynamics.

### 4.4. Future Work

Based on the results of the current study, our subsequent studies will focus on testing the technique on a larger number of healthy subjects and patients, with the aim of obtaining a criterion that differentiates normal changes in the type of vascular tone from pathological changes.

Future studies will focus on the changes in the type of vascular tone and the *PWV* during the different phases of breathing in healthy subjects.

The changes in the type of vascular tone were transmitted to the other limb (non-massaged), but, due to the nature of the experiment, we could not determine the time at which this transmission began, so a study must be carried out that records the signals during massage in one limb only and monitors the changes in vascular tone in the opposite limb. This may explain the nature of the transmission caused by different kinds of vascular tone regulation.

## 5. Conclusions

In this work, an evaluation of the information that can be obtained by tracking specific vascular tone changes using bioimpedance signals was made. The study was applied to a subject who was diagnosed with varicose veins in the lower extremities in the early stages. The signals from the lower extremities were recorded before and after applying a local massage to one of the extremities for five minutes. The obtained results confirm that the use of bioimpedance signals can provide quantitative and qualitative information about the amount of improvement in blood circulation and the type of vascular tone before and after applying a massage. We recommend using this method for monitoring in the event of applying other types of treatment, whether physical or pharmacological.

## Figures and Tables

**Table 1:** General data about recorded signals before and after massage.

	Record Duration (min)	Average Heart Rate (PPM)	Heart Cycle Count	Respiratory Rate (Cycle/min)	Inhalation Cycle Count	Exhalation Cycle Count
1—Before massage	3.04	61.8	188	9.5	55	66
2—After massage	2.07	61.35	127	8.9	37	49

**Table 2:** Change in the number of hypertonic cycles before and after massage.

	Before Massage				After Massage			
Segment	HC (B)	HT (B)	NT(B)	HT/HC (B) %	HC (M)	HT (M)	NT(M)	HT/HC (M) %
LLU	188	43	145	22.8%	127	3	124	2.4%
RLU	188	91	97	48.4	127	14	113	11%

## Data Availability

Hammoud, Ahmad (2022), “Bioimpedance signals recorded from the lower limbs of a patient with early-stage varicose veins before and after massage,” Mendeley Data, V1, https://doi.org/10.17632/frjj7djfpv.1.
